# Connecting the αα-hubs: same fold, disordered ligands, new functions

**DOI:** 10.1186/s12964-020-00686-8

**Published:** 2021-01-06

**Authors:** Lasse Staby, Katrine Bugge, Rasmus Greve Falbe-Hansen, Edoardo Salladini, Karen Skriver, Birthe B. Kragelund

**Affiliations:** REPIN, Department of Biology, University of Copenhagen, Copenhagen, Denmark

**Keywords:** Interactome, Intrinsically disordered protein, IDP, NCBD, PAH, RST, HHD, Signaling, TAFH, Hub proteins

## Abstract

**Background:**

Signal fidelity depends on protein–protein interaction–‘hubs’ integrating cues from large interactomes. Recently, and based on a common secondary structure motif, the αα-hubs were defined, which are small α-helical domains of large, modular proteins binding intrinsically disordered transcriptional regulators.

**Methods:**

Comparative structural biology.

**Results:**

We assign the harmonin-homology-domain (HHD, also named the harmonin N-terminal domain, NTD) present in large proteins such as harmonin, whirlin, cerebral cavernous malformation 2, and regulator of telomere elongation 1 to the αα-hubs. The new member of the αα-hubs expands functionality to include scaffolding of supra-modular complexes mediating sensory perception, neurovascular integrity and telomere regulation, and reveal novel features of the αα-hubs. As a common trait, the αα-hubs bind intrinsically disordered ligands of similar properties integrating similar cellular cues, but without cross-talk.

**Conclusion:**

The inclusion of the HHD in the αα-hubs has uncovered new features, exemplifying the utility of identifying groups of hub domains, whereby discoveries in one member may cross-fertilize discoveries in others. These features make the αα-hubs unique models for decomposing signal specificity and fidelity. Using these as models, together with other suitable hub domain, we may advance the functional understanding of hub proteins and their role in cellular communication and signaling, as well as the role of intrinsically disordered proteins in signaling networks.

**Video Abstract**

## Introduction

**Fig. 1:**
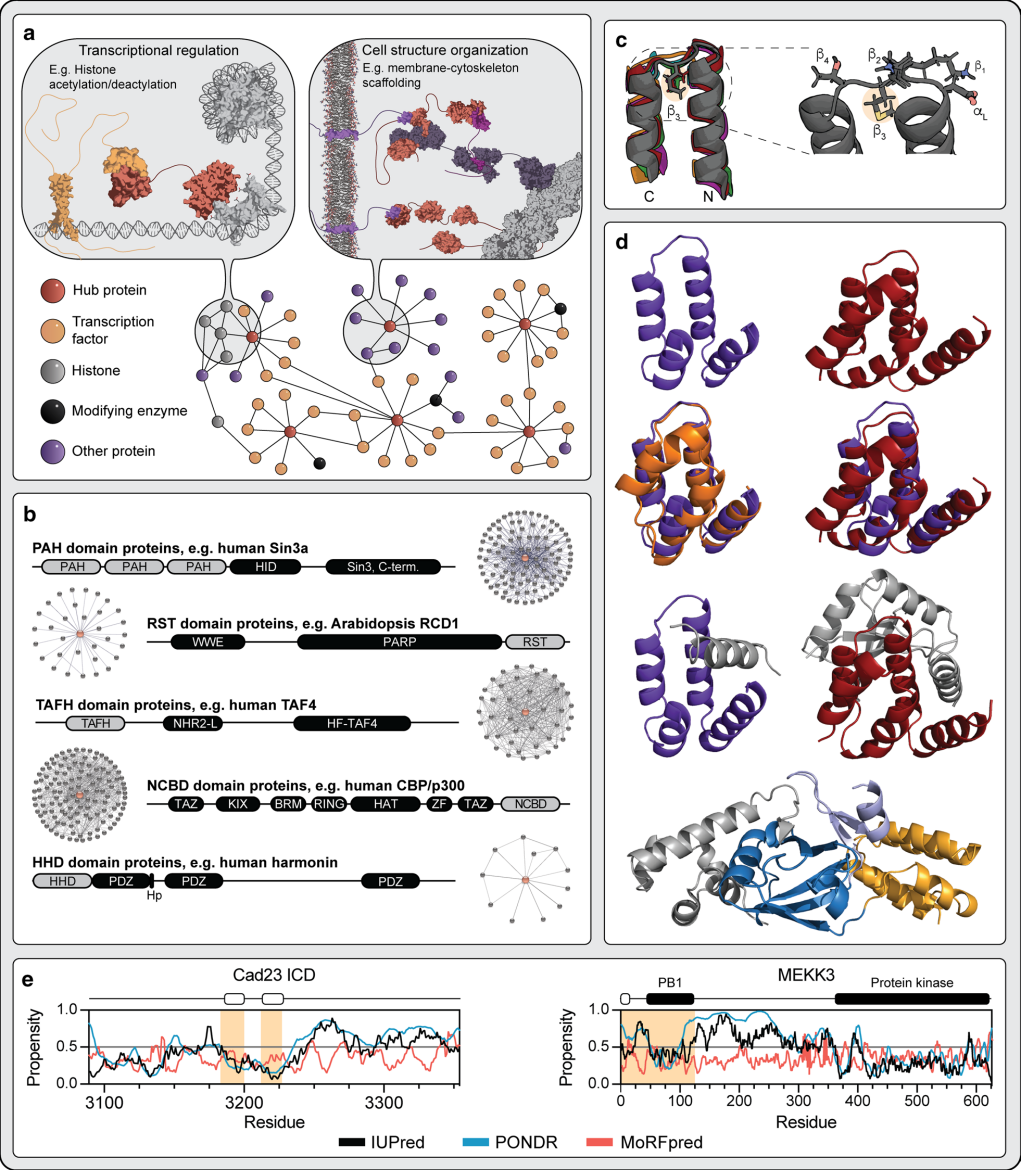
Connecting the αα-hubs. **a** Hub proteins are involved in diverse functions such as transcription, post-translational modification and organization of cell structure. Hub proteins are shown in the center of protein–protein interaction networks where they organize relevant molecular components. The drawings are based on αα-hub protein functions such as transcriptional regulation (Sin3a) and cell structure organization (harmonin). **b** Domain structure of αα-hub proteins. Representative parent protein (not drawn to scale) for each of the founding αα-hubs PAH, RST, TAFH and NCBD, as well as the new member HHD. Experimentally-based interactome sizes obtained from the STRING database are shown for each αα-hub. Hp: small hairpin extension of the HHD-PDZ supramodule. **c** Superimposition of the αα-hairpin super-secondary structure motif. Representatives of each domain type of the αα-hubs (pdb codes 2CZY (PAH1), 2LD7 (PAH3), 5ECJ (TAFH), 2L14 (NCBD), 2KBQ (HHD), 5N9Q (RST)). The zoom illustrates the α_L_-β_4_ link motif found in the prototypical αα-hubs [1], where the highlighted hydrophobic β_3_-anchor residue forms stabilizing interactions between the two hairpin helices. **d** Cartoon structures comparing αα-hubs. The PAH1 domain of Sin3a (purple) is compared to the HHDs of CCM2 (red), whirlin (dark orange) and harmonin (bright orange). Top row illustrates the two domains in their free form whereas the second row shows overlays of free PAH1 with whirlin HHD (left) and CCM2 HHD (right) (pdb codes 2RMR (PAH1), 6FDD (whirlin-HHD), 4FQN (CCM2-HHD)). The third row illustrates complexes of the two domains: PAH1 in complex with SAP25 and CCM2 HHD in complex with MEKK3 (pdb codes 2RMS and 4Y5O, respectively). The ligands are shown in grey. The bottom panel displays the harmonin PDZ-HHD supramodule (PDZ in blue) in complex with sans (grey). The short hairpin extension responsible for tethering the PDZ and HHD is shown in light blue. **e** Disorder profiles of HHD binding ligands cadherin-23 intracellular domain (ICD) and MEKK3. The disorder propensity ranging from 0 to 1 was predicted using IUPred2A (black) [32] and PONDR VSL2 (blue) [33], whereas MoRFs were predicted by MoRFpred (red) [34]. The regions responsible for binding to HHD based on available structures (pdb codes 4Y5O, 2LSR and 2KBR) are highlighted with orange background, whereas the regions binding as α-helix in the hydrophobic cleft are highlighted with white boxes

Fast and efficient regulation of cellular signaling is key to cell viability. Fidelity in signaling is mediated by large networks of interacting proteins linked by a few highly connected proteins called hubs (Fig. 1a). As a consequence, protein interaction networks are highly sensitive to the removal of hubs, which may lead to premature fatality [2, 3]. Accordingly, hub proteins are often subject to gene duplication resulting in functional redundancy that may protect against hub failure [4, 5]. Protein intrinsic disorder (ID) provides adaptability and is critical for hub functionality [6, 7]. Indeed, hub proteins are longer and have higher degree of ID than non-hubs [8], and they can be distinguished by specific sequence features of importance to their network evolution [9]. A detailed functional characterization of hub networks including both hubs and their binding partners revealed augmented disorder-enrichment in hub interactions among disease-associated proteins [10]. Nonetheless, folded hubs also exist, in which case structural disorder is found in the hub-partners. Thus, whereas a disordered hub, such as p53, can use different disordered regions for partner binding, a structured hub, such as 14-3-3, can associate with many different intrinsically disordered partners [11]. Folded hubs can also exist in large regulatory and modularly built proteins, and similarly, they bind numerous intrinsically disordered partners [12] using intriguing allosteric mechanisms [13], and constitute widespread cellular hubs of key biological relevance.

Bugge, Staby et al. [1] recently determined the structure of the RCD1, SRO and TAF4 (RST) domain from the plant protein Radical Induced Cell Death1 (RCD1), revealing a previously undescribed fold of four helices (H1–H4) forming an exposed hydrophobic binding cleft. However, similar folds have been determined for the PAH (paired amphipathic helix), TAFH (TATA-box-associated factor homology), and NCBD (nuclear coactivator binding domain) domains of the important human transcriptional regulators Sin3, transcription initiation factor TFIID-subunit 4 (TAF4) and CREB binding protein (CBP), respectively (Fig. 1b). These proteins have large interactomes and function as hubs. They have a common structural foundation consisting of an αα-hairpin super-secondary motif linked by an α_L_β_4_-loop, which serves as an organizing platform for malleable helices of varying length and topology (Fig. 1c). Hence, they were designated the αα-hubs. The prototypical αα-hub consists of four α-helices (although an additional helix can be present as in e.g. TAFH), of which two are organized in the αα-hairpin stabilized by the β_3_-loop residue acting as a hydrophobic anchor (Fig. 1c). As hubs, they bind multiple intrinsically disordered partners with pleiotropic functional outcomes, and their parent proteins are large modular proteins implicated in transcription (Fig. 1b).

In addition to the initially identified αα-hubs, we find, based on structural analyses (Fig. 1d), that the harmonin-homology domain (HHD), present in the proteins whirlin [14], harmonin [15], cerebral cavernous malformations 2 (CCM2) [16] and regulator of telomere elongation 1 (RTEL1) [17], also has the prototypical traits of αα-hubs [1, 17]. Therefore, we here connect the HHD to the αα-hubs. However, similar to NCBD, it lacks β_3_-anchoring between H2 and H3, and instead has an additional α-helix H5 positioned between H3 and H4, similar to TAFH. Still, its αα-hairpin superimpose perfectly with those of the other αα-hubs (Fig. 1c), while its overall architecture is most similar to the PAH1 domain (RMSD of 0.85 Å for H1-H4 of whirlin HHD; Fig. 1d). Like the other αα-hub-harboring proteins, the HHD-containing proteins are large, modular proteins, but they have entirely different biological functions, related to hearing-vision perception for whirlin and harmonin [14, 15], neurovascular integrity for CCM2 [16] and telomere length regulation for RTEL1 [17] (Fig. 1a, b).

The known αα-hub ligands are mostly disordered in their free state [18, 19], typically interacting via short linear motifs [20–22]. These are of similar chemical compositions with hydrophobic and acidic residues as key hotspots for the interactions, and typically undergo coupled folding and binding to form an α-helix when bound in the hydrophobic cleft of the αα-hub [1]. Like the other αα-hubs, the currently characterized HHD complexes involving MEKK3 and cadherin-23 primarily use the common hydrophobic cleft for binding of an amphipathic α-helix [16]. Of note, the binding regions of HHD ligands are located in longer predicted disordered regions. As an example, the region of MEKK3 that directly binds to the typical αα-hub binding cleft of the HHD of CCM2 has been predicted to be intrinsically disordered in the free state (7) (Fig. 1e). ID also characterizes the αα-hub binding regions of the well-characterized PAH domain ligands repressor element 1 silencing transcription factor (REST) [23] and Mad1 [24], and RST ligands DREB2A and ANAC013 [20]. Similar to PAH, the ligand-binding cleft is in HHD located between helix H1 and H2.

With the inclusion of HHD, new features are added to the αα-hubs. The HHD of harmonin tethers the neighboring PDZ domain via a small hairpin extension of PDZ1 to form a functional and structurally stable supramodule responsible for binding the protein Sans as part of hearing-vision regulation [15] (Fig. 1b, d). Binding of the hairpin extension occurs through a surface different from the α-helix-binding groove, a feature similarly seen for HHD of CCM2 (Fig. 1d). Here, the CCM2-HHD uses both the α-helix-binding cleft between H1 and H2 and a large surface on the backside of H2 and H3 for binding of MEKK3 [16] (Fig. 1d). Despite taking advantage of several surface areas simultaneously for ligand binding, the known harmonin interactome appears much smaller than for the other αα-hubs (e.g. Sin3a has > 100 experimentally identified partners vs. 11 in harmonin) (Fig. 1b).

Some of the new functional features arising from the inclusion of HHD in the αα-hubs can retrospectively be found in the literature for αα-hubs and vice versa. Thus, it is likely that the tandem HHDs of RTEL1, linked by a proliferating cell nuclear antigen (PCNA) interacting protein (PIP) box [25], forms a supramodular function and protein-interaction platform in RTEL1 [17]. Furthermore, the PAH domains may also exist as supramodules, as a Sin3a fragment of PAH1 and PAH2 interacts more strongly with the transcription factor Mad1/Mxd1 than the isolated domains [2]. The PAH3 domain of Sin3a binds the histone deacetylase complex subunit Sin3-associated protein (SAP30) in a high affinity complex resulting from cooperative recognition of two discrete surfaces of PAH3 by the tripartite binding motif in SAP30 [26]. NCBD binds not only disordered proteins in complicated mechanisms [27, 28], but also folded partners [29, 30], and similarly, HHD of CCM2 binds the folded PB1 region of MEKK3 [16] (Figure D). Thus, the αα-hubs are highly versatile and can adapt to many different partners, although intrinsically disordered ligands remain a highly dominant common feature. These findings highlight the importance of identifying groups of similar domains, whereby discoveries in one member may cross-fertilize discoveries in others, advancing their understanding. Furthermore, as a group, the αα-hubs have the potential to foster progress in the understanding of signal fidelity and specificity governed by other small α-helical hub domains, such as KIX and TAZ from CBP [12], for which such highly divergent functions have not yet been observed.

The simple fold of the αα-hubs and the consistent appearance of small α-helix domains in transcriptional regulation and signaling, and in particular in hubs [1, 12], suggest them to be of optimal architecture for integration via signals embedded in structural disorder. For the αα-hubs it is tempting to hypothesize that the expanded interaction surface and the cooperativity associated with supramodular structures, coupled to structural malleability, form a highly versatile platform for affinity- and specificity-tuning. This could ensure signal fidelity across networks. Indeed, the harmonin N-terminal HHD-containing supramodule bound a fragment of Sans consisting of the two domains SAM and PBM with a *K*_d_ of ~ 1 nM (Fig. 1d), which was three and four orders of magnitude lower than the *K*_d_s for binding the isolated SAM and PBM domains [15]. Whether other αα-hubs also exploit supramodular structures or if they use other interaction surfaces than the hydrophobic cleft, remains to be systematically addressed. So far, the limited focus on “one domain—one binding site” has precluded answering these questions and jointly, the αα-hubs leave more open questions than answers. In fact, many other signaling domains exist, which like the αα-hubs, function as hubs but with different structural features [31]. Connecting hub domains using the approach presented here will have the prospect to cross-fertilize studies beyond the single hub protein, and provide new insight not directly available from one hub on its own.

## Conclusion

With the inclusion of HHD in the αα-hubs, we wish to put focus on the αα-hubs as an attractive model system for scrutinizing underlying mechanisms of signal fidelity and specificity, as well as communication by structural disorder within the cell. This is necessary to forward the understanding of key features of cellular hubs, including how interactome sizes are determined. Connecting the hubs, as done here, opens for exciting new research questions addressing properties across the domains, inspired by individual members. The approach used to connect hubs, is directly applicable to other folded hubs. For the αα-hubs, the inclusion of a new member will foster a new focus on structural disorder and its role in HHD linked interactomes.

## Data Availability

All data generated or analyzed during this study are included.

## Abbreviations

CBP: CREB binding protein
CCM2: Cerebral cavernous malformations 2
HDD: Harmonin homology domain
MEKK3: Mitogen activated kinase kinase kinase 3
ID: Intrinsic disorder
NCBD: Nuclear coactivator binding domain
PAH: Paired amphipathic helix
PCNA: Proliferating cell nuclear antigen
RCD1: Radical induced cell death1
REST: Repressor element 1-silencing transcription factor
RST: RCD1, SRO and TAF4
TAFH: TATA-box-associated factor homology
RTEL1: Regulator of telomere elongation 1
